# *Lacticaseibacillus rhamnosus* TR08 alleviated intestinal injury and modulated microbiota dysbiosis in septic mice

**DOI:** 10.1186/s12866-021-02317-9

**Published:** 2021-09-18

**Authors:** Jiangtao Yin, Wen Sun, Xianqiang Yu, Xiaojia Xiao, Baiqiang Li, Zhihui Tong, Lu Ke, Wenjian Mao, Weiqin Li

**Affiliations:** 1 Department of Critical Care Medicine, Jinling Hospital of Nanjing Medical University, 305 East Zhongshan Road, Nanjing, 225001 China; 2 Department of Critical Care Medicine, Affiliated Hospital of Jiangsu University, Zhenjiang, China; 3 Department of Critical Care Medicine, Jurong Hospital Affiliated to Jiangsu University, Zhenjiang, China; 4 Southeast University School of Medicine, Nanjing, China; 5 Department of Critical Care Medicine, Jinling Hospital, Medical School of Nanjing University, Nanjing, China

**Keywords:** *Lacticaseibacillus rhamnosus* TR08, Sepsis, Intestinal mucosa, Gut microbiota

## Abstract

**Background:**

Probiotics are widely used in intestinal microbiota imbalance caused by sepsis, however, the protective mechanism is still unclear. This study aimed to explore protective effect of *Lacticaseibacillus rhamnosus* TR08 on intestinal injury in septic mice.

**Results:**

The levels of serum inflammatory factors were reduced significantly in septic mice treated with *L. rhamnosus* TR08. The levels of sIgA in terminal ileum were significantly higher in probiotic treatment group than sepsis group. Intestinal pathological damage in septic mice improved and the expression of tight junction proteins increased after probiotic treatment. Sequencing of fecal microbiota showed that the abundance and diversity of probiotic treatment group were significantly better than those of sepsis group, and beneficial bacteria increased while some bacteria decreased in the phylum level.

**Conclusion:**

*L. rhamnosus* TR08 could improve the integrity of intestinal barrier, enhance the intestinal mucosal immunity in septic mice, and rebalance the intestinal microecosystem.

## Background

Sepsis is one of the important problems in critical care medicine [1]. More than 19 million people suffer from sepsis every year worldwide, with very high fatality rate [2, 3]. Approximately 3 million of patients who survive after sepsis have cognitive impairment [4]. Despite antibiotic treatment and surgical intervention, mortality rate of sepsis remains high [5]. Sepsis can be diagnosed when the sequential organ failure assessment (SOFA) rises by 2 points or more from the baseline in patients with infection or suspected infection [6]. Sepsis is mainly secondary to burn, severe acute pancreatitis and pneumonia. The intestine is the largest immune organ in human and gastrointestinal dysfunction occurs when sepsis affects the intestine, such as feeding intolerance, gastrointestinal bleeding, and paralytic intestinal obstruction [7]. It is reported that sepsis leads to the imbalance of intestinal microecosystem including the reduction of microbiota diversity and abundance [8].

Probiotics are a kind of active microorganisms that are beneficial to the host by colonizing in the human body and changing the composition of microbiota, with the potential to maintain the integrity of intestinal mucosa, reduce bacterial translocation and prevent infection [9, 10]. Clinical studies and meta-analysis demonstrated that probiotics could reduce the risk of infection in critically ill patients [11, 12]. Probiotics could promote the recovery of gastrointestinal motility by regulating the production of T lymphocytes in the host intestine and reducing the secretion of inflammatory factors in intestinal epithelial cells [13, 14]. Moreover, probiotics increased the level of immunoglobulin A secreting cells in the lamina propria of intestinal mucosa, promoted the secretion of secreted immunoglobulin A (sIgA) to maintain intestinal homeostasis [15].

However, the mechanisms of probiotic-mediated protection against sepsis are poorly understood. In the development of sepsis, microecological imbalance has been regarded as a key feature. Therefore, our primary hypothesis is that *Lacticaseibacillus rhamnosus TR08* could improve integrity of the intestinal barrier and enhance intestinal mucosal immunity, and the second hypothesis is that *L. rhamnosus TR08* could rebalance the intestinal microecosystem. In this study we evaluated the effects of *L. rhamnosus* TR08 on inflammation, intestinal mucosal barrier, intestinal microbiota using mouse model of sepsis.

## Results

### *L. rhamnosus* TR08 improved the integrity of the intestinal barrier

**Fig. 1:**
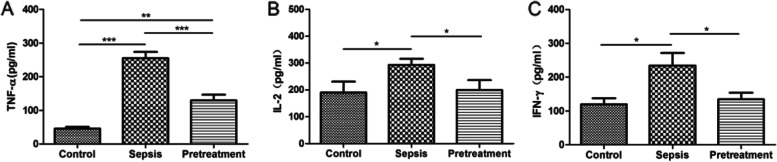
*L. rhamnosus* TR08 pretreatment reduced systemic inflammation in septic mice. The levels of TNF-α (**A**), IL-2 (**B**) and IFN-γ (**C**) were significantly higher in sepsis group compared with control group, but decreased significantly after pretreatment. *P < 0.05; **P < 0.01; ***P < 0.001

ELISA showed that the levels of TNF-α, IL-2 and IFN-γ were significantly higher in sepsis group compared with control group (*P* < 0.05). After pretreatment with probiotics, their levels were significantly lower than in sepsis group (*P* < 0.05) (Fig. 1). These data indicated that *L. rhamnosus* TR08 improved the integrity of the intestinal barrier in septic mice.

### *L. rhamnosus* TR08 promoted the secretion of sIgA

**Fig. 2:**
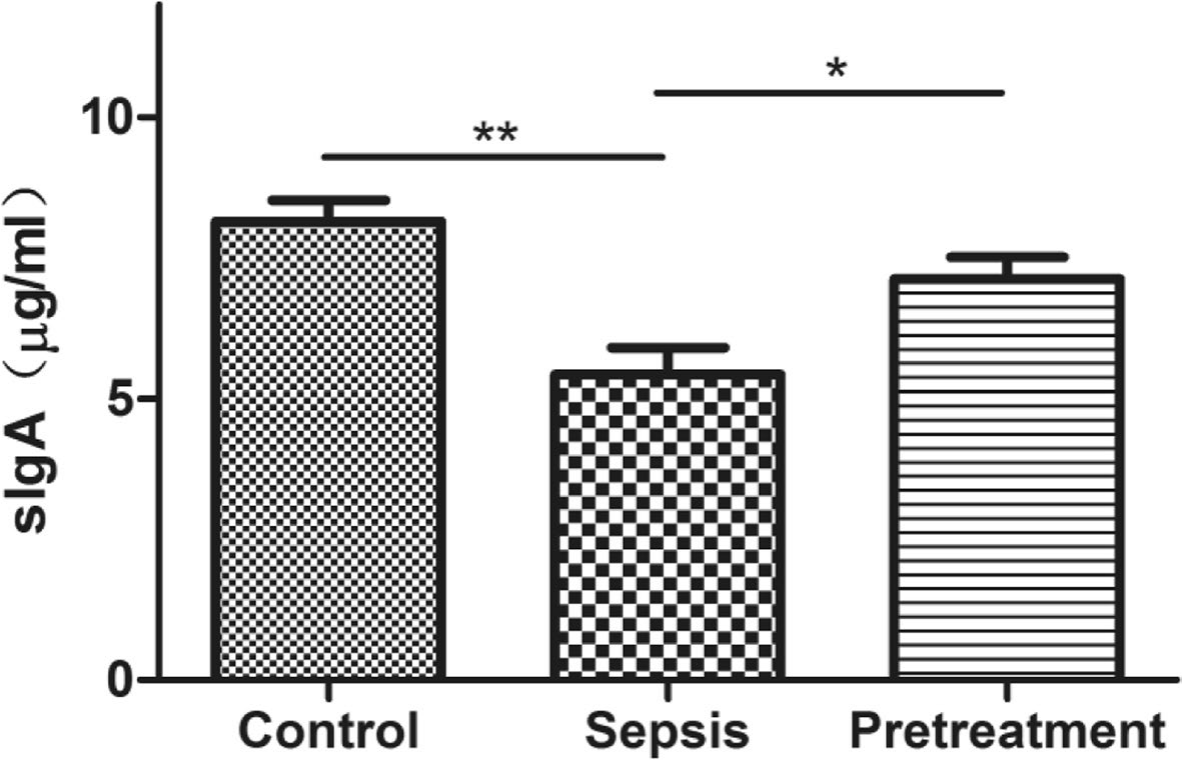
*L. rhamnosus* TR08 pretreatment promoted the secretion of sIgA in septic mice. The level of sIgA in mucosal tissue of the terminal ileum in sepsis group significantly reduced compared with control group, and increased after pretreatment. *P < 0.05; **P < 0.01

ELISA showed that the level of sIgA in mucosal tissue of the terminal ileum in sepsis group was significantly lower compared with control group (*P* < 0.01). However, sIgA level of pretreatment group was significantly higher than in sepsis group (*P* < 0.05) (Fig. 2). These results indicated that pretreatment with *L. rhamnosus* TR08 could improve mucosal immunity of septic mice.

### *L. rhamnosus* TR08 relieved intestinal pathological damage

**Fig. 3:**
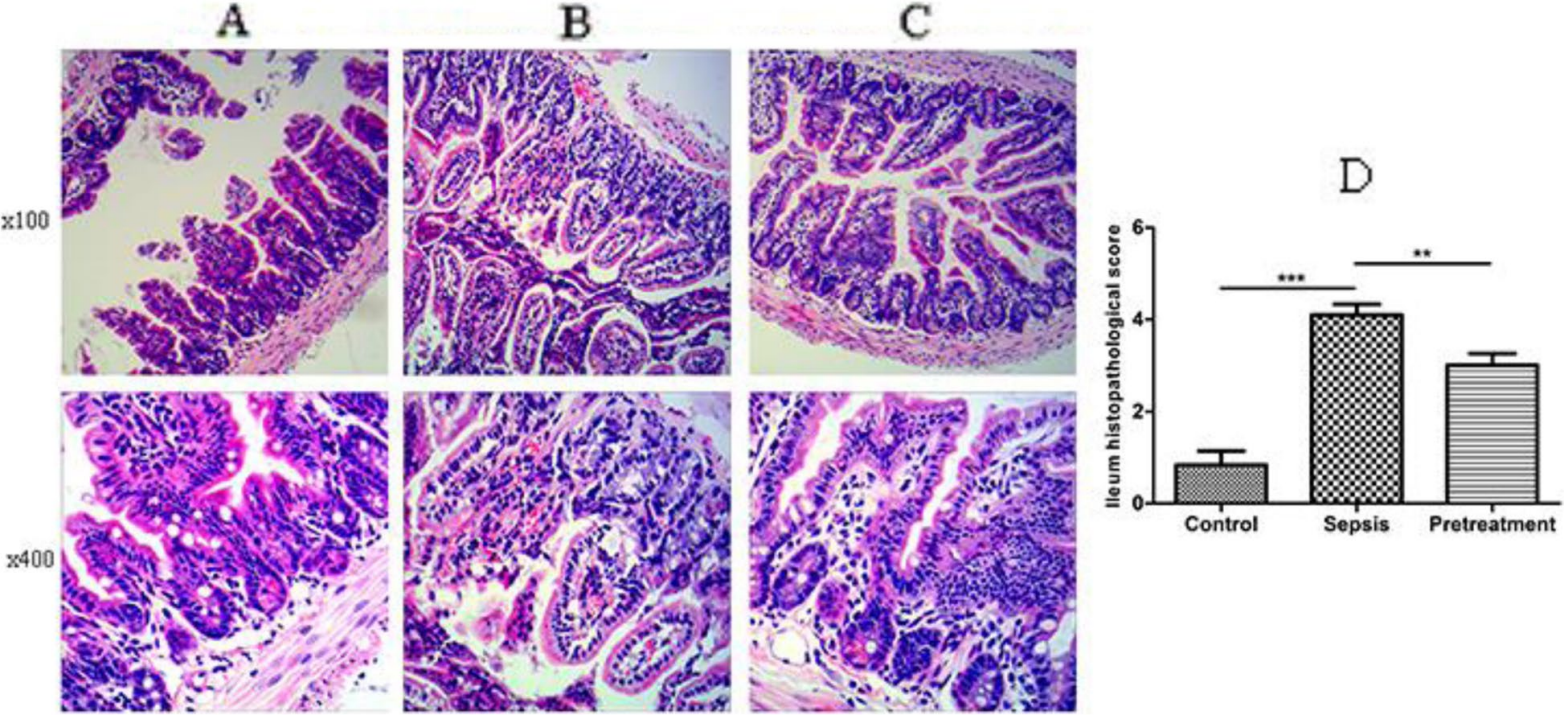
*L. rhamnosus* TR08 pretreatment attenuated the injury of terminal ileum mucosa in septic mice. **A** The structure of pithelial tissue and smooth muscle of the terminal ileum was complete in control group. **B** The structure of mucosal epithelial tissue and smooth muscle layer was destroyed, the capillaries were congested or even ruptured, accompanied by inflammatory cell infiltration in septic mice. **C** The intestinal mucosal epithelial structure was complete without erosion, villi and glands were arranged normally, and the smooth muscle layer structure was clear. **D** The intestinal injury score in sepsis group significantly elevated compared with control group, and decreased after pretreatment. **P < 0.01; ***P < 0.001

Pathological changes of terminal ileum tissue were evaluated by hematoxylin and eosin (H&E) staining. In control group, the epithelial tissue of the terminal ileum was smooth, the villi and glands were arranged neatly, and the structure of smooth muscle was clear (Fig. 3). Moreover, there was no infiltration of inflammatory cells. However, in sepsis group the intestinal mucosal epithelial tissue was destroyed, the villi and glands were arranged disorderly and deformed, and the capillaries in the structure were congested or even ruptured. Furthermore, the smooth muscle layer structure was disordered, and accompanied by inflammatory cell infiltration. Interestingly, the intestinal injury of mice in pretreatment group was significantly improved compared to sepsis group. The intestinal mucosal epithelial structure was complete without erosion, villi and glands were arranged normally, and the smooth muscle layer structure was clear. In addition, there was few inflammatory cells infiltration (Fig. 3A-C). Subsequently, quantitative pathological scores of the terminal ileum were calculated. The intestinal injury score in sepsis group significantly elevated compared with control group (*P* < 0.001), and decreased after probiotics pretreatment (*P* < 0.01) (Fig. 3D).

### *L. rhamnosus* TR08 improved intestinal mucosal permeability

**Fig. 4:**
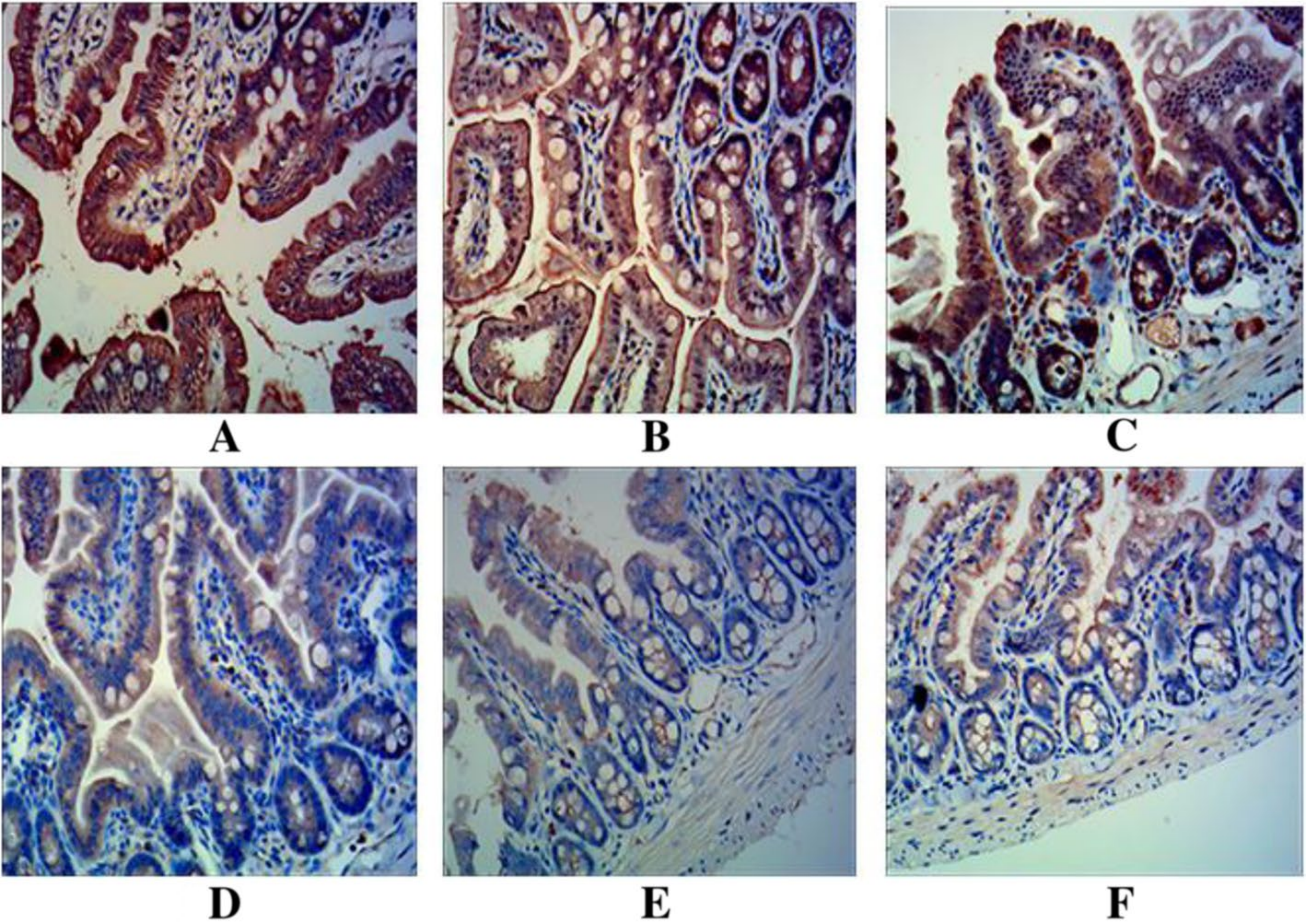
*L. rhamnosus* TR08 pretreatment improved intestinal mucosal permeability in septic mice. The occludin and ZO-1 proteins in the nucleus, cell membrane and cytoplasm were stained brown and distributed evenly (**A**, **D**). The staining in sepsis group was significantly lighter than control group (**B**, **E**), and was stronger than sepsis group after pretreatment with *L. rhamnosus* TR08 (**C**, **F**). **A**, **B**, **C** expression of occludin; **D**, **E**, **F** expression of ZO-1

To examine the effect of *L. rhamnosus* TR08 on the permeability of intestinal mucosa, we detected the expression of tight junction proteins occludin and ZO-1 in three groups of mice. Occludin and ZO-1 proteins were stained brown and distributed evenly (Fig. 4A, D). The staining of occludin and ZO-1 proteins was significantly lighter in sepsis group than that of control group (Fig. 4B, E), and was stronger in pretreatment group than that of sepsis group but lighter than that of control group (Fig. 4C, F).

**Fig. 5:**
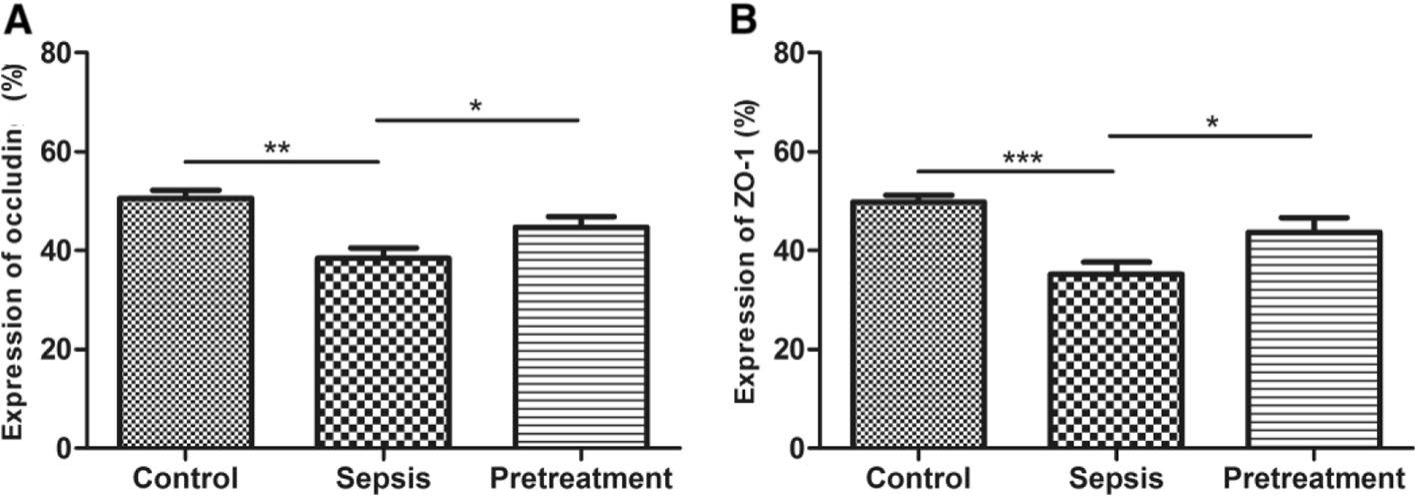
Quantification of the immunohistochemistry results. **A** The expression of occludin. **B** The expression of ZO-1. *P < 0.05; **P < 0.01; ***P < 0.001

Quantitative analysis demonstrated that the staining intensity of occludin protein was significantly lower in sepsis group than in control group (*P* < 0.01), but was significantly higher in probiotics pretreatment group (*P* < 0.05) (Fig. 5A). Similarly, the staining intensity of ZO-1 protein was significantly lower in sepsis group than in control group (*P* < 0.001), but was significantly higher in probiotics pretreatment group (*P* < 0.05) (Fig. 5B).

### *L. rhamnosus* TR08 increased microbial abundance and diversity

**Fig. 6:**
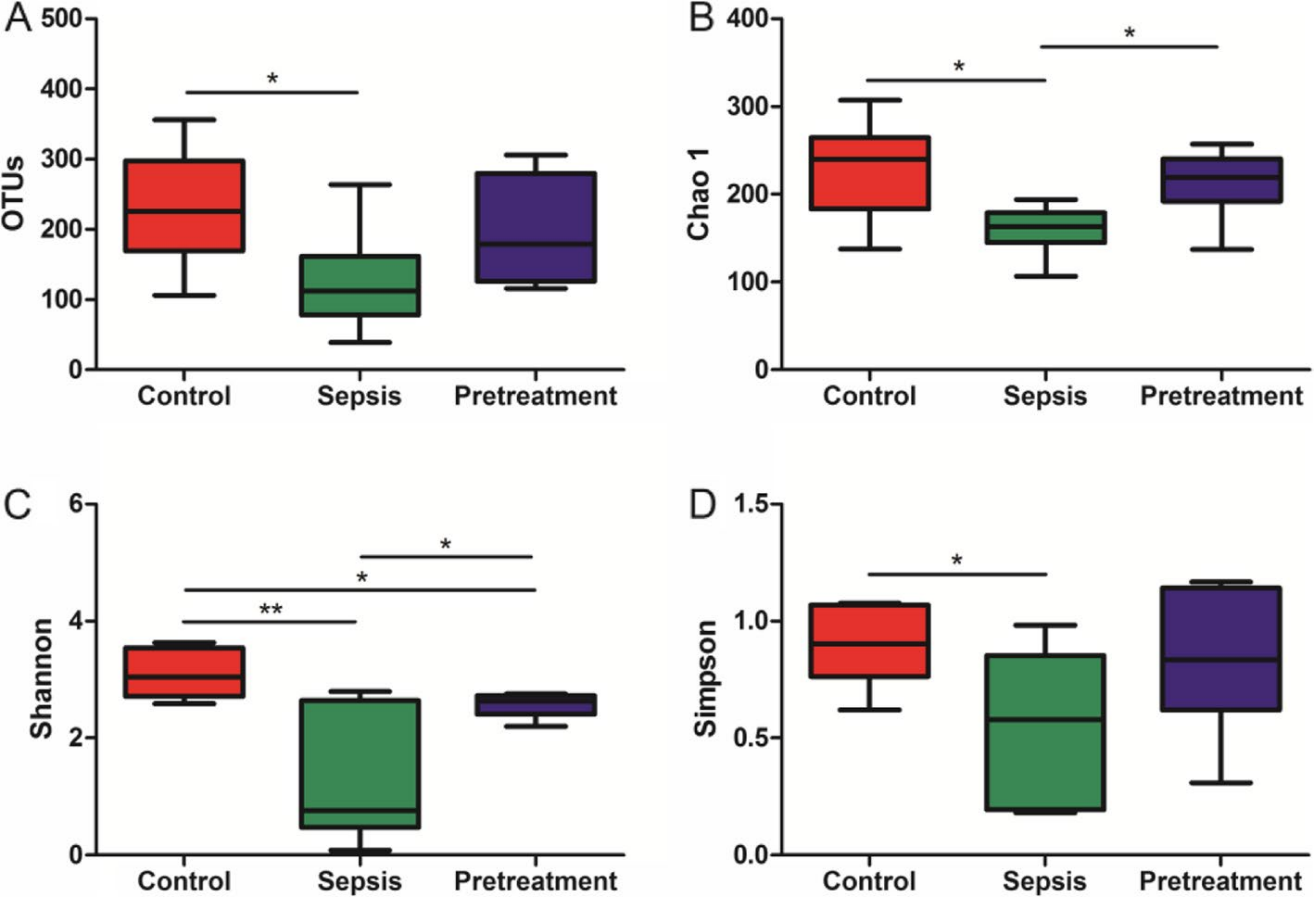
*L. rhamnosus* TR08 increased gut microbial abundance and diversity of septic mice. **A** Number of OTUs. **B** Abundance index (Chao 1). **C**, **D** Diversity index (Shannon and Simpson). OTUs: operational taxonomic units. *P < 0.05; **P < 0.01

The coverage index was above 97% per sample, indicating that the number of sequences we tested was sufficient to represent most of intestinal microorganisms in the sample. The operational taxonomic units (OTUs), abundance index (Chao 1) and diversity index (Shannon and Simpson) were significantly lower in sepsis group than in control group (*P* < 0.05), and the indices of Chao 1 and Shannon increased after probiotics pretreatment (*P* < 0.05) (Fig. 6). These results indicated that sepsis reduced the abundance and diversity of gut microbiota, but could be reversed by pretreatment with *L. rhamnosus* TR08.

#### Principal coordinates analysis of the three groups

**Fig. 7:**
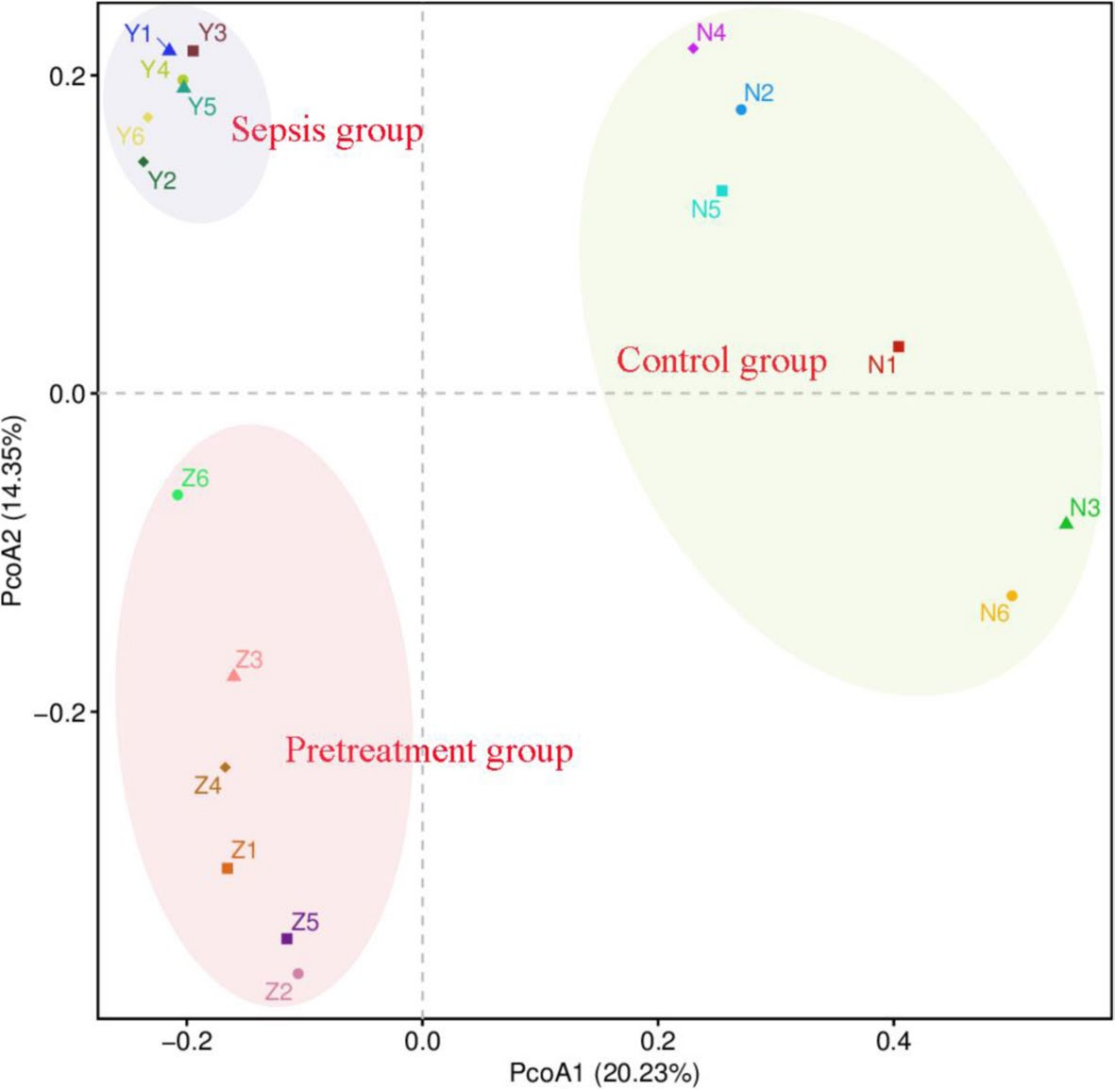
PCoA of intestinal microbiota. Microbial community structure in the three groups was separated apparently

Principal coordinates analysis (PCoA) is a method to examine the similarity or difference of data. Therefore, we utilized PCoA plot to determine the phylogenetic similarities between gut microbiota in three groups. Microbial community structure in the three groups was different (Fig. 7).

#### Analysis of the composition of intestinal microbiota in mice

**Fig. 8:**
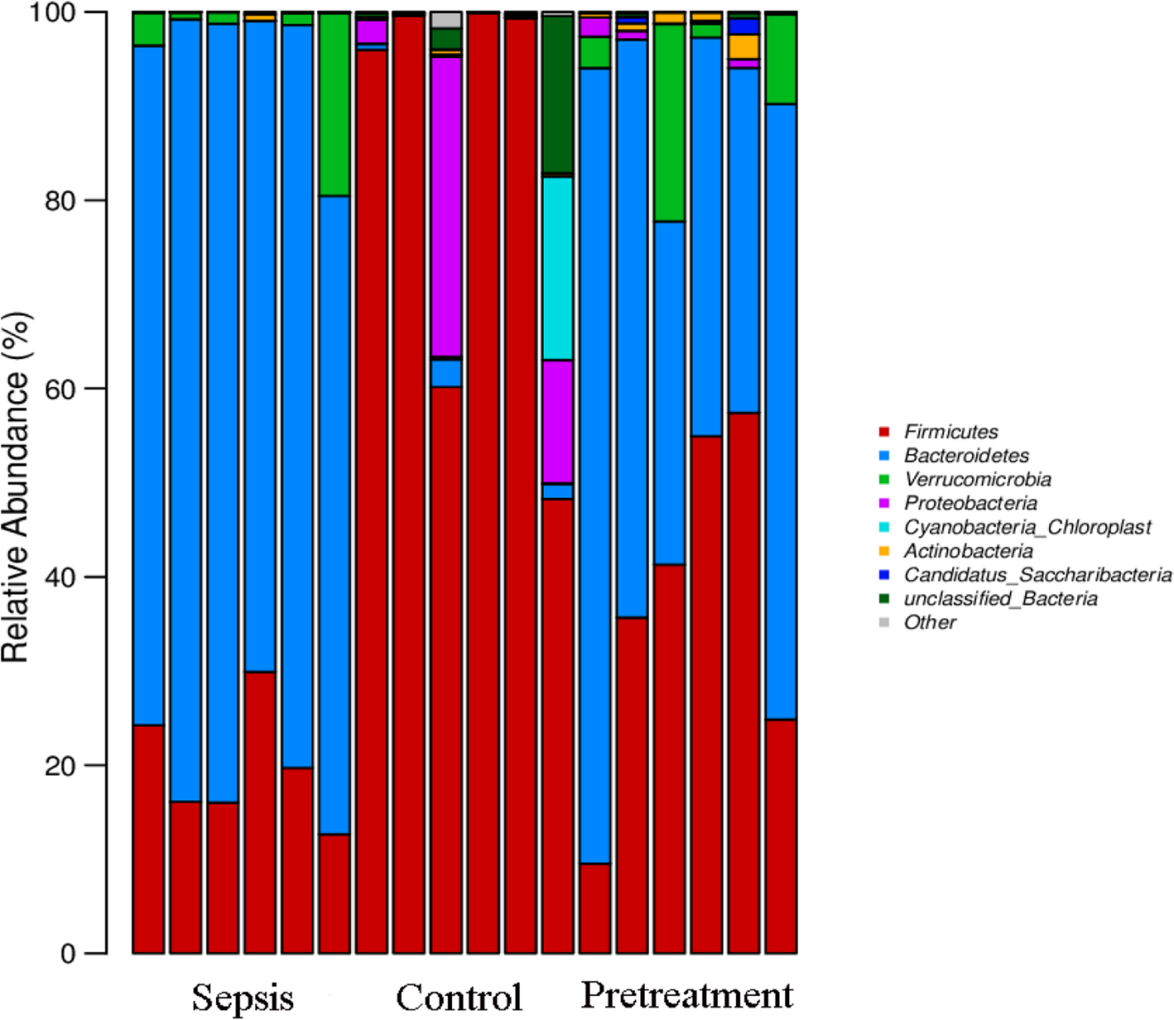
Histogram of the composition of intestinal microbiota in phylum level

**Fig. 9:**
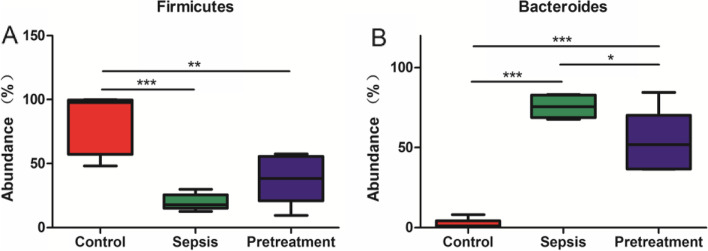
Analysis of the composition of intestinal microbiota of the three groups of mice in phylum level. There were differences in the phylum level of Firmicutes and Bacteroides. **A** The abundance of Firmicutes of sepsis group significantly reduced compared with control group and increased after probiotics pretreatment. **B** The abundance of Bacteroides of sepsis group significantly increased compared with control group and decreased after pretreatment

There were 22 different microbiota at the phylum level (Fig. 8). Firmicutes, Proteobacteria, and Cyanobacteria were the dominant phyla in control group, accounting for 83.87, 7.99 and 3.36%, respectively. Bacteroidetes, Firmicutes and Verrucomicrobia were the dominant microbiota in sepsis group, accounting for 75.65, 19.77 and 4.35%, respectively. The dominant microbiota of pretreatment group were the same as sepsis group, accounting for 54.45, 37.29 and 5.88%, respectively. There were differences in the phylum level of Firmicutes and Bacteroides among the three groups. The abundance of Firmicutes was significantly lower in sepsis group compared to control group (*P* < 0.001), and was increased after probiotics pretreatment without statistical significance (*P* = 0.0503) (Fig. 9A). Moreover, the abundance of Bacteroides was significantly higher in sepsis group compared to control group (*P* < 0.001), and was decreased after probiotics pretreatment (*P* < 0.05) (Fig. 9B).

**Fig. 10:**
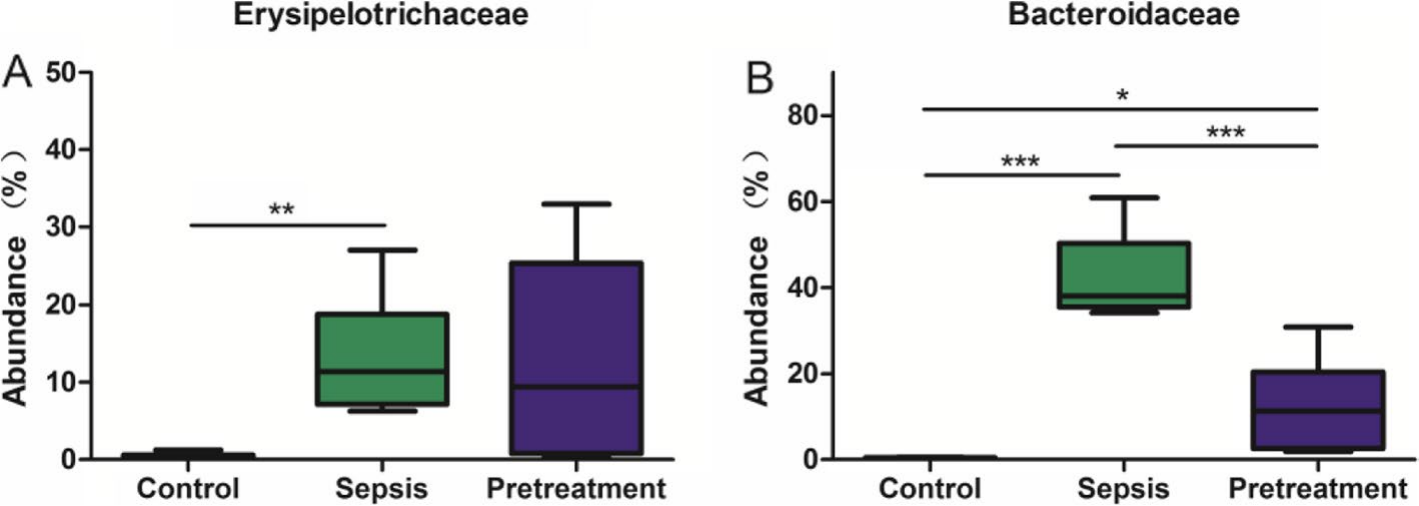
Analysis of the composition of intestinal microbiota of the three groups of mice in family level. There were differences in the phylum level of Erysipelotrichaceae and Bacteroidaceae. **A** The level of Erysipelotrichaceae of sepsis group increased compared with control group but without difference after probiotics pretreatment. **B** The level of Bacteroidaceae of sepsis group significantly increased compared with control group and reduced after pretreatment

There were 189 different microbiota at the family level. The abundance of Clostridiaceae, Peptostreptococcaceae and Chloroplast ranked the top three in control group, accounting for 78.38, 3.49 and 3.35%, respectively. The abundance of Bacteroidaceae, Porphyromonadaceae and Erysipelotrichaceae ranked the top three in sepsis group, accounting for 42.30, 15.73 and 13.24%, respectively. Furthermore, Porphyromonadaceae, Lactobacillaceae and Erysipelotrichaceae were the three most abundant bacterial families in pretreatment group, accounting for 28.75, 15.99 and 12.64%, respectively. We analyzed the microbiota with OTUs abundance > 0.1% and found significant difference in the family level of Erysipelotrichaceae and Bacteroidaceae. The level of Erysipelotrichaceae was significantly higher in sepsis group compared to control group (*P* < 0.01), but was not significantly decreased after probiotics pretreatment (Fig. 10A). The level of Bacteroidaceae was significantly higher in sepsis group compared to control group (*P* < 0.001), but was significantly reduced after probiotics pretreatment (*P* < 0.001) (Fig. 10B).

## Discussion

Probiotics are active microorganisms that colonize in human body and are beneficial to human by promoting nutrient absorption, regulating host mucosa and immune function, and keeping the balance of the intestinal microbiota [16, 17]. *L. rhamnosus* is a type of gram-positive, anaerobic, and acid-resistant probiotic present in the intestines [18, 19]. *L. rhamnosus* plays an important role in intestinal health by enhancing the survival of intestinal mucosal crypts, reducing intestinal epithelial cell apoptosis and maintaining the integrity of the cytoskeleton [20]. It was reported that *L. rhamnosus* could increase the secretion of Th1 cytokines in rat spleen cells, inhibit the secretion of Th2 cytokines, and inhibit the drift of Th1 to Th2, thereby enhancing cellular immune response [21]. *L. rhamnosus* TR08 used in this study was derived from the intestines of healthy adults.

Sepsis is a clinical syndrome resulting from the dysregulation of host response to a pathogen, which can be secondary to severe systemic inflammatory response syndrome (SIRS) and inflammatory damage [22, 23]. Our results showed that sepsis increased the production of inflammatory cytokines TNF-α, IL-2 and IFN-γ, which was ameliorated by pretreatment with *L. rhamnosus* TR08.

The intestine is one of the most vulnerable organs during sepsis [24]. Sepsis causes intestinal ischemia and hypoxia, destroys the structure of intestinal mucosa, produces a large number of metabolites and toxins, leading to intestinal microbiota disorder [25]. Moreover, intestinal antigen presenting cells are activated, intestinal mucosal barrier is undermined, and intestinal mucosal immunity is damaged [26]. Increased intestinal mucosal permeability, impaired intestinal mucosal barrier, and displacement of specific bacteria in intestinal will further aggravate sepsis [27]. In this study, pathological damage and the immunity of intestinal mucosa of septic mice were significantly improved after intervention with probiotics. Furthermore, the expression of tight junction protein in intestinal epithelial cells increased, indicating that the intestinal permeability was improved. These results suggest that *L. rhamnosus* TR08 could maintain the stability of intestinal mucosa structure effectively.

The intestinal homeostasis is disrupted in sepsis due to endogenous and exogenous factors, which is manifested as decreased diversity of intestinal microbiota, decreased abundance of beneficial bacteria, and increased abundance of specific bacteria [28]. It was reported that the number of dominant bacteria such as anaerobic bacteria in the gut decreased during sepsis, and pathogenic bacteria became dominant microbiota [29]. Intestinal microbiota imbalance in critically ill patients may be related to the use of antibiotics [30]. Sequencing results of our study showed that *L. rhamnosus* TR08 significantly increased the abundance and diversity of intestinal microbiota in septic mice. Moreover, the abundance of Firmicutes increased and the abundance of Bacteroides decreased after pretreatment with *L. rhamnosus* TR08 in septic mice. Firmicutes has high content of peptidoglycan in the cytoderm, which is generally regarded as a beneficial bacteria [31]. However, Bacteroides can cause infection when the immune function of the body is disturbed or the microbiota is imbalanced [32]. Our results indicated that *L. rhamnosus* TR08 pretreatment could increase the abundance of bacteria in the gut of septic mice.

However, our study has some limitations. First, we did not analyze the relationship between probiotic pretreatment and prognosis in septic mice, and we will analyze survival curves of mice in future experiments. Second, we did not use germ-free model. Third, the change in the number of intestinal microbes only considers 16 s rDNA measurement. Metabolomic mass spectrometry analysis of microbial metabolites will help validate the types of intestinal microbiota.

## Conclusion

In summary, we demonstrated that prophylactic *L. rhamnosus* TR08 therapy could improve the integrity of intestinal barrier, enhance the intestinal mucosal immunity in septic mice, and rebalance intestinal microecosystem.

## Methods

### Ethics statement

This study was carried out in compliance with the ARRIVE guidelines. All animal experiment protocols were approved by the Animal Care Ethics Committee of the Affiliated Hospital of Jiangsu University (Zhenjiang, China). Twenty-six 4-week-old male Kunming mice (weight 20 ± 2 g) were purchased from Jiangsu University and housed in pathogen-free animal facilities under a standard 12-h-light/12-h-dark cycle at room temperature with humidity of 50–60%.

### Probiotic administration and septic model

*L. rhamnosus* TR08 was provided by Zhenjiang Tianyi Health Co., Ltd. After 3 days of normal feeding, the mice in intervention group were intragastrically administered with 200 µl of *L. rhamnosus* TR08 at 10^9^ CFU/ml every day, the control group and the sepsis group were given the same amount of saline, and all three groups of mice were given intragastric administration for 4 consecutive weeks prior to modeling. Sepsis model was made by intraperitoneal injection of 10 mg/kg lipopolysaccharide (LPS) as described previously [33].

### ELISA

Blood samples were obtained from eyeball at 24 h post-modeling and centrifuged at 3,000 r/min at 4 °C for 15 min. The serum samples were stored at -70 °C until use. Serum levels of TNF-α, IL-2 and IFN-γ were evaluated using commercial ELISA kits (Boster, Wuhan, China) following the manufacturer’s protocols.

The terminal ileum was collected after decapitation, and cut into sections about 1 cm for homogenization. The lysates were centrifuged at 4,000 r/min at 4 °C for 15 min and supernatants were collected. sIgA levels in supernatants were determined using commercial ELISA kit (Boster, Wuhan, China) following the manufacturer’s protocols.

### Histological analysis

Terminal ileum tissues were collected from mice at 24 h post-modeling and fixed in 10% formalin. The fixed tissues were embedded in paraffin and sectioned at 4 µm thickness. The sections were stained with H&E for histological analysis and evaluated by the chiu's small intestine score system [34].

For immunohistochemistry staining for occludin and ZO-1, sections were incubated with 3% H_2_O_2_ at room temperature for 5 min to inactivate endogenous peroxidase, and then blocked with 5% bovine serum album in at 37˚C for 1 h. Next, the sections were incubated with rabbit anti-occludin and anti-ZO-1 polyclonal antibody (Hua'an Biotech, Hangzhou, China) at room temperature for 2 h, and incubated with secondary antibody at 37˚C for 1 h. Finally, the sections were washed and observed under an Olympus BX60 upright fluorescence microscope (Olympus, USA).

### Fecal microbiota analysis

Feces were collected from colon after decapitation. Total DNA in colon contents was extracted using a Mag-Bind Stool DNA Kit (Omega, Norcross, GA, USA). The 16 S rDNA gene amplicon library was constructed using barcoded universal bacterial primers (for the V3-V4 region of the bacteria 16 S rRNA gene):515 F (5’- GTGCCAGCMGCCGCGGTAA -3’) and 806 R (5’- GGACTACHVGGGTWTCTAAT -3’). AxyPrep DNA Gel Extraction Kit (Axygen Biosciences, Union City, CA, USA) and fluorometric kit (Quant-iT PicoGreen, Invitrogen, Carlsbad, CA, USA) were used for PCR, and all raw reads were screened using the Quantitative Insights Into Microbial Ecology software (QIIME, version 1.17). UCHIME was used to identify and remove chimeras. USEARCH was applied to generate OTUs, reads with the maximum length in each OTU were selected as representative sequences. Based on the bacterial SILVA dataset, representative sequences were assigned to different taxonomic levels. The diversity and richness of gut microbiota were detected by indexes chao, simpson and Shannon, respectively. Differences among groups were analyzed by PCoA.

### Statistical analysis

Statistical analysis was performed using SPSS 22.0 (Chicago, IL, USA). All results were expressed as mean ± standard deviation (SD). Comparisons across groups were analyzed using one-way analysis of variance and Least Significant Difference. For microbiome analysis, Principal-component analysis and Random forest were conducted using the web-based tool MicrobiomeAnalyst 4.0 (www.microbiomeanalyst.ca). Two-tailed non-parametric Kruskal–Wallis test was used to compare the differences in diversity indexes and microbial taxa. A *P* < 0.05 was considered significant.

## Data Availability

All data and material are included in this manuscript.

## Abbreviations

LPS: Lipopolysaccharide
OTUs: Operational taxonomic units
PCoA: Principal coordinates analysis
SD: Standard deviation
SIgA: Secreted immunoglobulin A
SIRS: Systemic inflammatory response syndrome
SOFA: Sequential organ failure assessment
